# Identification, expression, and endocrine-disruption of three ecdysone-responsive genes in the sentinel species ***Gammarus fossarum***

**DOI:** 10.1038/s41598-018-22235-7

**Published:** 2018-02-28

**Authors:** D. Gouveia, F. Bonneton, C. Almunia, J. Armengaud, H. Quéau, D. Degli-Esposti, O. Geffard, A. Chaumot

**Affiliations:** 1 Irstea, UR RiverLy, Laboratoire d’écotoxicologie, centre de Lyon-Villeurbanne, 5 rue de la Doua CS 20244, F-69625 Villeurbanne, France; 2 Laboratoire Innovations technologiques pour la Détection et le Diagnostic (Li2D), Service de Pharmacologie et Immunoanalyse (SPI), CEA, INRA, F-30207 Bagnols sur Cèze, France; 3 IGFL, Université de Lyon, CNRS UMR5242, Ecole Normale Supérieure de Lyon, Université Claude Bernard Lyon 1, 46 allée d’Italie, F-69364 Lyon, France

## Abstract

Taking advantage of a large transcriptomic dataset recently obtained in the sentinel crustacean amphipod *Gammarus fossarum*, we developed an approach based on sequence similarity and phylogenetic reconstruction to identify key players involved in the endocrine regulation of *G*. *fossarum*. Our work identified three genes of interest: the nuclear receptors RXR and E75, and the regulator broad-complex (BR). Their involvement in the regulation of molting and reproduction, along with their sensitivity to chemical contamination were experimentally assessed by studying gene expression during the female reproductive cycle, and after laboratory exposure to model endocrine disrupting compounds (EDCs): pyriproxyfen, tebufenozide and piperonyl butoxide. RXR expression suggested a role of this gene in ecdysis and post-molting processes. E75 presented two expression peaks that suggested a role in vitellogenesis, and molting. BR expression showed no variation during molting/reproductive cycle. After exposure to the three EDCs, a strong inhibition of the inter-molt E75 peak was observed with tebufenozide, and an induction of RXR after exposure to pyriproxyfen and piperonyl butoxide. These results confirm the implication of RXR and E75 in hormonal regulation of female reproductive cycles in *G*. *fossarum* and their sensitivity towards EDCs opens the possibility of using them as specific endocrine disruption biomarkers.

## Introduction

Endocrine disrupting compounds (EDCs) are exogenous substances that interfere with hormone-regulated physiological processes and provoke adverse health effects in exposed organisms and/or their progeny^1^. EDCs typically interfere with hormone signaling, acting as agonist or antagonist, or with hormone synthesis, through anti-hormonal effects for example^2,3^. These compounds constitute a worldwide concern for potential human health implications^4^, especially due to the multiple developmental and reproductive disorders observed in wildlife^5–9^.

In aquatic environments, occurrence and biological effects of EDCs have been reported, such as masculinization and feminization events. This has led to the need of specific monitoring and the development of several robust, specific and reliable biomarkers of EDC exposure in vertebrates, such as the vitellogenin (Vtg) induction in male fishes^10–12^. However, due to the molecular divergence acquired through animal evolution, vertebrate-derived biomarkers cannot be used in invertebrates, which represent the majority of animals and key species for ecosystem functioning. The need to develop relevant tools for identification and assessment of EDCs toxic effects and mode of action in invertebrates has been already highlighted by the scientific community. The discovery of imposex in marine gastropods due to Tributyltin (TBT)^13^, and intersex occurrence in natural populations of gammarids^14,15^ are two examples of the importance of developing more studies of EDCs effects in reproductive function of invertebrate species. Nevertheless, due to a currently limited knowledge in invertebrate endocrinology, today there is still a scarce understanding of EDCs modes of action in the majority of invertebrate species.

Among crustacean species, the amphipod *Gammarus fossarum* is commonly used as a sentinel species in freshwater risk assessment^16^. Many toxicity markers are available in this species, mainly based on life-history traits such as reproductive features^17^, locomotor behavior/feeding rate^18^, neurotoxicity^19^ and genotoxicity^20^. The recent advances in proteomics techniques led to the development of a specific methodology based on liquid chromatography coupled to tandem mass spectrometry for quantifying a Vtg protein in *G*. *fossarum*^21^. However, the proposal of this protein as an endocrine disruption (ED) biomarker for male feminization was discarded after laboratory and field experiments that yielded high variability and low Vtg inductions in organisms exposed to contaminants^22^. Similar conclusions were reached when analyzing expression levels of two Vtg genes in *Echinogammarus marinus*, highlighting the fact that invertebrate biomarkers must be developed and validated *de novo*, and not just derived from their eukaryotic counterparts^23^. More recently, a proteogenomic study based on the alliance of transcriptomic and proteomic approaches^24^ lead to the construction of a database (GFOSS database) containing 1,873 experimentally validated *Gammarus fossarum* specific-proteins^25^. However, few proteins involved in hormonal regulation could be recognized from this database, and potential key candidates for ED biomarkers were not identified. Indeed, such proteins are expected to be present at very low levels and therefore are hence probably lost in the “background noise” of the proteome.

**Figure 1:**
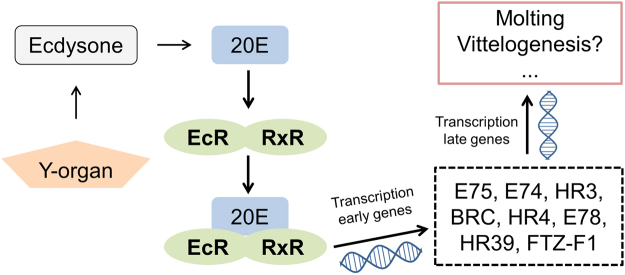
Putative ecdysone regulatory cascade in crustaceans. Ecdysone is secreted by the Y-organ, leading to its active form 20-hydroxyecdysone (20E). The heterodimer EcR/RXR receives 20E and initiates the transcription of the “early” response genes, which in turn regulates the transcription of “late” genes responsible for initiating molting and vitellogenesis. Modified from^30^.

Taking this context into account, we hypothesized that a gene candidate approach considering a thorough bioinformatics mining of the transcriptomic database obtained in *G*. *fossarum* could be used to identify candidate biomarkers of endocrine disruptor effects in this species. As discussed in previous studies^17,26^, the reproduction cycle of female amphipods such as gammarids (vitellogenesis/oogenesis, embryogenesis) is closely correlated with molting. Disruption of these processes by contamination results in reproductive impairment. In crustaceans, molting is regulated by a multi-hormonal system including specific neuropeptide hormones, juvenoid hormones such as methyl farnesoate (MF), and biological active forms of ecdysteroids such as 20-hydroxyecdysone (20E) or ponasterone A (reviewed in^26^ and^3^). Among arthropods, while the ecdysone pathway is well characterized in insects, much less is understood in crustacean species. In insects, 20E binds to and activates the ecdysone receptor/retinoid-X-receptor (EcR/RXR, mainly known as USP in insects) nuclear receptor complex, initiating a cascade of gene-regulatory events that will mediate molting and reproduction (Fig. 1). Early response genes from the insect ecdysone pathway include the nuclear receptors ecdysone induced protein 75B (E75), ecdysone induced protein 78 C (E78) and hormone receptor 3 (HR3), and the transcription factors broad-complex (BR) and ecdysone induced protein 74EF (E74)^27^. In crustaceans, early response genes are thought to be similar to those of insects^3,28,29^.

The present study aimed at identifying gene candidate biomarkers of ED effects in the crustacean *G*. *fossarum*. Assuming a possible homology between crustacean and insect endocrine regulator genes, we mined the transcriptomic GFOSS database and performed sequence similarity searches and phylogenetic analysis in order to identify some key players potentially involved in endocrine regulation in *G*. *fossarum*. Since the ecdysone pathway is crucial for arthropod molting and reproduction, our work focused on ecdysone-responsive genes whose homologous sequences were identified in GFOSS. Through molecular phylogenetic analysis, we validated the annotations of the nuclear receptors E75 and RXR, and of the transcription factor BR in *G*. *fossarum* sequences. In order to obtain reliable nucleotide sequences of gene candidates and primers for subsequent gene expression studies, these genes were cloned and sequenced. Their involvement in the endocrine control of molting and reproductive process and sensitivity to EDCs were evaluated by studying their profile during the female reproductive cycle and following laboratory exposure to three chemicals acting on the hormonal regulation of target arthropods: pyriproxyfen, tebufenozide and piperonyl butoxide.

## Results and Discussion

### Phylogenetic sequence validation

In the present study, based on the GFOSS RNAseq-derived transcriptome database, we aimed to identify *G*. *fossarum* specific sequences homologue to key genes known to be involved in endocrine regulation of arthropods. Seeing the importance of ecdysone pathway in arthropod molting and reproduction, we focused on three ecdysone-responsive genes: RXR, EcR and BR. After blast sequence similarity searches against GFOSS, the top three results were kept for phylogenetic validation of their annotations. As nuclear receptors are paralog genes with a highly evolutionary conserved domain called DNA-binding domain (DBD) and other conserved amino-acyl sequences in functional areas in the ligand-binding domain (LBD), the BLAST searches for RXR and EcR sequences yielded common results (Table 1). Sequences GFOSS_83504 (annotated as ultraspiracle - same as RXR - in GFOSS) and GFOSS_2900 (annotated as nuclear hormone receptor E75 in GFOSS database) were found among the top three blast results of both searches using the query sequences of RXR and EcR from the decapod *Eriocheir sinensis* and the amphipod *Hyalella azteca*, respectively. Concerning BR, blast results yielded one sequence GFOSS_1452 (annotated as broad-complex isoform Z2) and two unassembled reads seq. 986730_fr4 and seq. 1061041_fr1.

**Table 1:** Sequence similarity search results after blastp of RXR, EcR and BR query sequences against the GFOSS database.

Query sequence (Accesion n° - name - [species])	Top 3 blast results	Score	Identitites	Percentage	Expect	GFOSS annotation
XP_018017941.1 PREDICTED: ecdysone receptor-like isoform X1 [Hyalella azteca]	GFOSS_83504_fr5	94	39/86	45	1,0E-18	ultraspiracle
	GFOSS_2900_fr4	70	95/389	24	1,0E-11	Nuclear hormone receptor E75
	seq. 406923_fr4	70	30/47	63	2,0E-11	—
XP_018008501.1 PREDICTED: broad-complex core protein isoforms 1/2/3/4/5-like isoform X4 [Hyalella azteca]	GFOSS_1452_fr3	281	162/230	70	7,00E-75	broad-complex, isoform Z2
	seq. 986730_fr4	276	132/139	94	2,00E-73	—
	seq. 1061041_fr1	274	132/139	94	7,00E-73	—
AHF65151.1 retinoid X receptor [Eriocheir sinensis]	seq. 307974_fr2	228	113/198	57	3,0E-59	—
	GFOSS_83504_fr5	159	71/90	78	2,0E-38	ultraspiracle
	GFOSS_2900_fr4	114	81/307	26	1,0E-24	Nuclear hormone receptor E75

**Figure 2:**
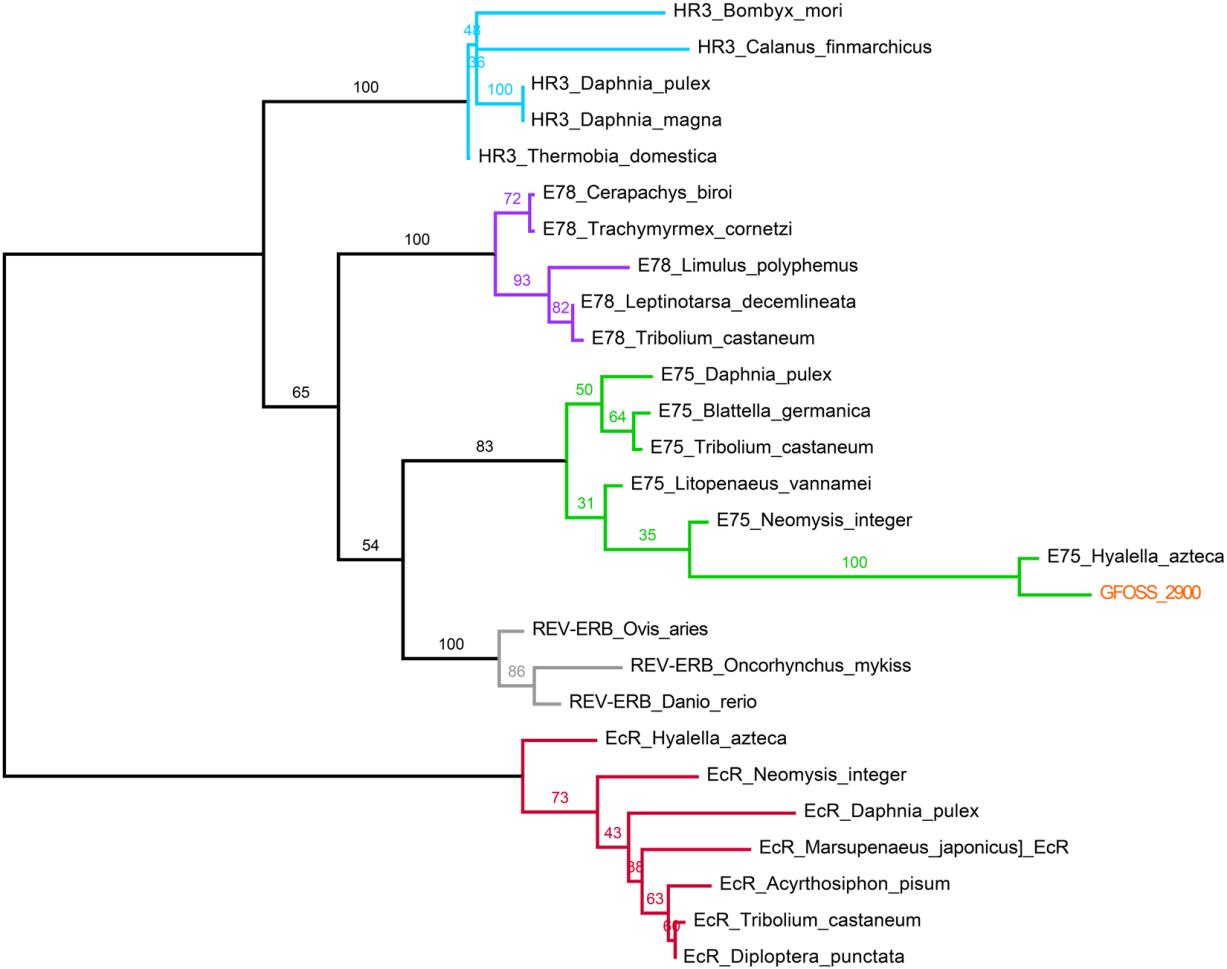
Phylogenetic tree of the GFOSS_2900 sequence. Tree construction was based on a homologous sequence dataset constructed using ortholog and paralog sequences from the same multigenic family as EcR (nuclear receptors E78, E75 and HR3).

**Figure 3:**
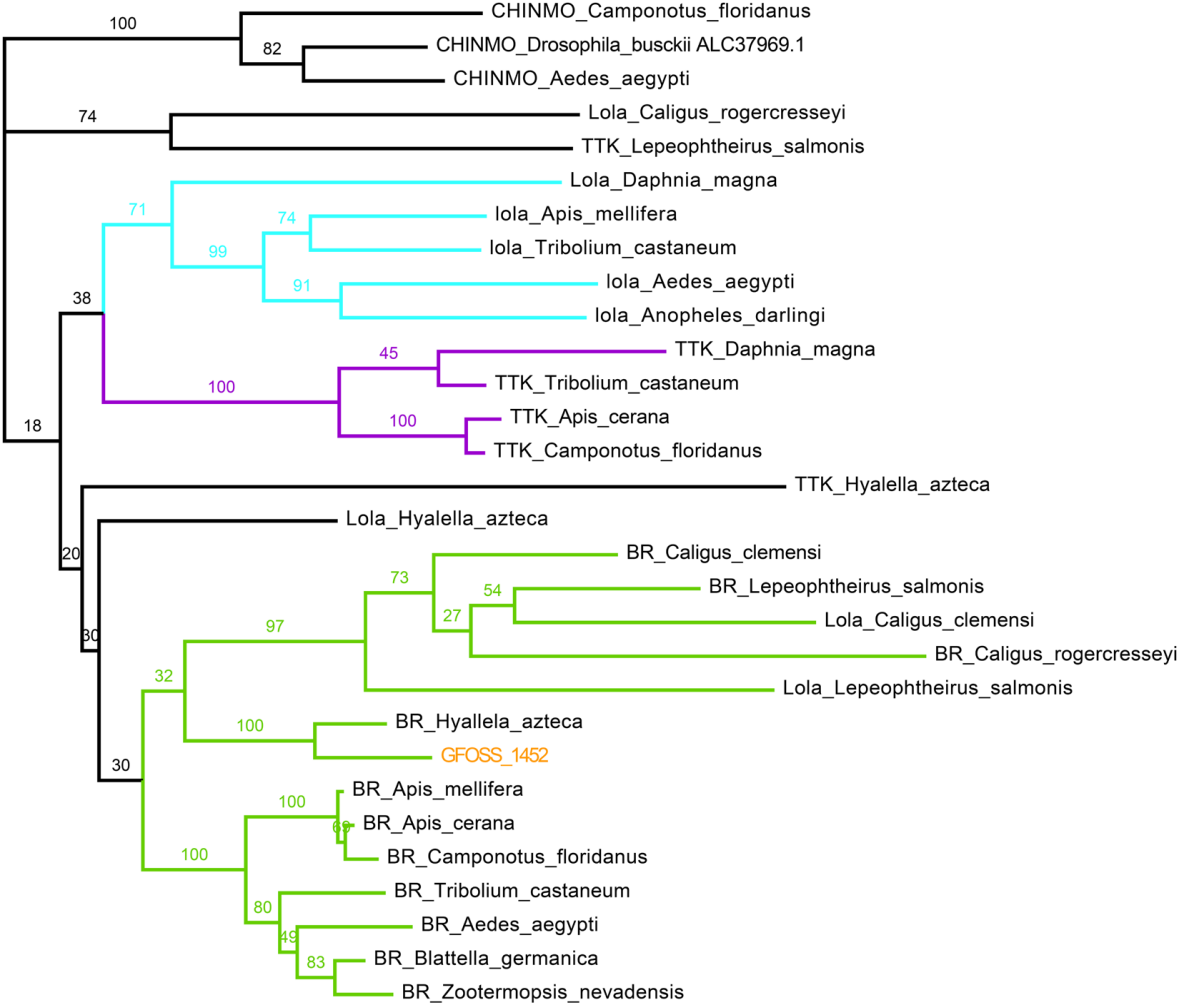
Phylogenetic tree of the GFOSS_1452 sequence. Tree construction was based on a homologous sequence dataset constructed using ortholog and paralog sequences from the same multigenic family as BR (transcription factors tramtrack TTK and longitudinals-like protein lola). CHINMO was used as an outgroup.

**Figure 4:**
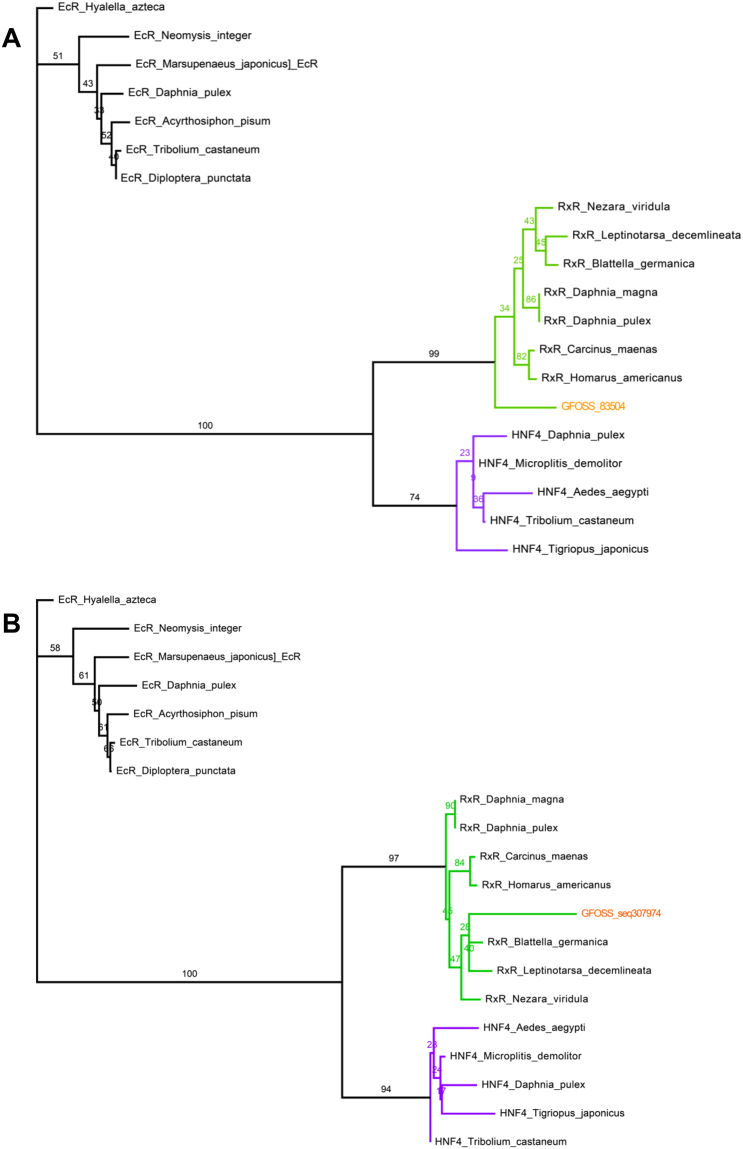
Phylogenetic tree of the (**A**) GFOSS_83504 sequence and (**B**) GFOSS_seq. 307974 read. Tree construction was based on a homologous sequence dataset constructed using ortholog and paralog sequences from the same multigenic family as RXR (nuclear receptor HNF4). EcR was used as an outgroup.

The sequence annotations available in GFOSS (reported in Table 1) were previously generated by an automatic bioinformatic pipeline based on reciprocal blast procedure against public sequence databases^25^. Such approach can be flawed due to affectation of functional annotations from a paralog sequence to the gene identified in the species of interest. Then, in order to select the *G*. *fossarum* ortholog genes of RXR, EcR, and BR, phylogenetic analyses were conducted to validate the annotation of the candidate sequences. Each *G*. *fossarum* sequence from Table 1 was thus aligned with ortholog sequences from different crustacean/insect species, and paralog sequences from different members of the corresponding multigenic family, *i*.*e*. nuclear receptor family for RXR and EcR, and BTB-ZF family for BR (sequence accession numbers are listed in Supplementary Table 1). The phylogenetic trees for candidate sequences are represented in Fig. 2 (EcR), Fig. 3 (BR) and Fig. 4 (RXR).

As presented in Fig. 2, sequence GFOSS_2900 did not cluster with EcR sequences, but rather with sequences from the nuclear receptor E75, confirming the bioinformatic functional annotation on GFOSS. The unassembled read GFOSS_seq. 406923 (47 amino acids long) also clustered with E75 sequences (data not shown) and therefore it also does not correspond to a *G*. *fossarum* EcR sequence. As expected, both these *G*. *fossarum* sequences showed the closest relationship with E75 from *Hyalella azteca*, the only genome-sequenced crustacean amphipod. These results show that no EcR transcripts were sequenced in the GFOSS RNAseq-derived transcriptome database. Because seq. 406923 is a short non-assembled read prone to have sequence errors, it was not used for primer design and sequence amplification. However, the E75 annotated sequence GFOSS_2900 was kept for subsequent studies. E75 is in fact one of the primary target genes of the EcR/RXR heterodimer, and regulates further downstream transcriptional cascades for molting and reproductive processes in both insects and decapod crustaceans^30–32^.

Figure 3 shows the phylogenetic tree obtained from the sequence dataset constructed for BR. CHINMO, TTK and lola genes from crustaceans and insects were considered to build this tree for the BTB-Zf family. As expected, the *G*. *fossarum* sequence GFOSS_1452 clustered together with the BR sequence from *H*. *azteca*, along with the other BR branches for crustaceans and insects. Similarly to the EcR results, the unassembled reads seq. 986730 and seq. 1061041 were not considered for the subsequent studies. Of note, this tree evidences that available annotation of crustacean sequences from this BTB-Zf family could be questioned due to potential duplication events unfound in the insects, and which apparently may have led to misannotations. For example, lola genes from *Caligus clemensi* and *Lepeophteirus salmonis* clustered with the crustacean BR branch. This means that this specific family of genes is still relatively unknown and poorly described in crustacean species, and some of the annotations available by successive sequence similarity derivation when annotating new genomes could be erroneous. However, given the good blast score obtained and the posterior phylogenetic analysis for sequence GFOSS_1452, we validated the BR annotation for this sequence.

In Fig. 4 are represented two phylogenetic trees, one for each RXR *G*. *fossarum* sequence identified by blast. Two sequences were retained for this gene in order to obtain a higher alignment length. In fact, sequence GFOSS_83504 covers only the DBD domain of the RXR gene, while the unassembled read GFOSS_seq. 307974 covers another part of the gene, the ligand-binding domain (LBD). Analyzing the trees, both sequences clustered with the RXR branch, and were thus validated as being RXR sequences.

RXR, E75 and BR were successfully cloned and sequenced using the primers from Table 2. For E75, a 419 nucleotide sequence was obtained, covering a portion of both the DBD and LBD of this nuclear receptor. Smaller sequences were obtained for BR and RXR (278 and 220 nucleotides, respectively). The 220 nucleotide RXR sequence corresponds to primers designed in the DBD domain, using only the GFOSS_83504 sequence. Other primer pairs were tested using forward sequences on the GFOSS_83504 and reverse sequences on seq. 307974, in order to obtain a sequence covering both DBD and LBD. However, we failed to amplify the RXR sequence with the pairs of primers tested.

**Table 2:** Nucleotide sequences of the primers used in the PCR and qPCR experiments.

	Primer ID	5′ - > 3′ nucleotide sequence	Fragment (bp)	Ct standard deviation	
				Experiment 1	Experiment 2
Target genes	RXR FW1	AAACCTTTGTGCCATCTGTG	220	–	–
	RXR RV3	GAACAGCCTCCCTCTTCATG			
	BRC FW1	GGGAGCAGACCAACAGTTCT	278	–	–
	BRC RV2	TGACCTCGCCATGGTACATA			
	E75 FW1	TCGTCAATGCAGCAAGAATC	419	–	–
	E75 RV2	GGATGTTCTTGGCGAAGGT			
Reference genes	EF FW	TTCAAGTATGCCTGGGTGCT	82	0,42	0,34
	EF RV	CGAACTTCCAGAGAGCAATGTC			
	GADPH FW	CGCTGGCCAGAACATCATTC	–	0,84	0,25
	GADPH RV	CGGCCTTGATGTCGTCGTAA			
	18 S FW	TGGGGGAGGTAGTGACGAAATC	–	0,81	0,71
	18 S RV	CCTGCGCTCGATACAGACATTC			

### Expression during female reproductive cycle

**Figure 5:**
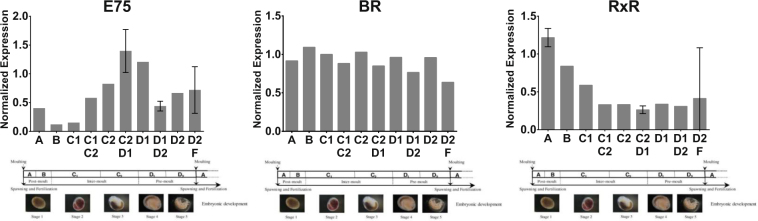
Expression profiles of E75, BR and RXR during the female reproductive cycle. Expression values of target genes were normalized to the expression of the reference gene EF. Each bar is the mean obtained from three technical replicates. Each condition (A, B, C1, C1/C2, C2, C2/D1, D1, D1/D2, D2 and D2 F) contains mRNAs from five individuals pooled together. The represented standard deviations derive from the individual analysis of the five biological replicates (Supplementary Figure 2) from the selected stages.

In order to assess the possible involvement of the three ecdysone-inducible genes in reproductive processes, we quantified their expression during the female reproductive/molt cycle. The bar graphs in Fig. 5 represent the expression levels of the three genes through the different stages. E75 and RXR transcripts showed important modulations during the female reproductive cycle, while BR remained relatively stable.

### E75

The expression pattern of E75 presents a strong peak at the C2/D1 stage, with a fold change of 12 relative to stage B. The C2 developmental stage is characterized by the quick increase in yolk formation and oocyte size, namely the start of secondary vitellogenesis^17,33^. This continuous increase from the C1/C2 until the C2/D1 stage suggests that the expression of E75 could be related to the onset of secondary vitellogenesis and oogenesis. This expression peak was validated through the analysis of individual replicates, where statistically significant difference was observed between C2D1 and D1D2 stages (*p* value < 0.0001, Supplementary Figure 2). A second slight increase in the levels of this transcript in the final D2 stage (Fig. 5) could indicate the existence of a second short peak of expression, as observed by the individual-specific increases obtained in biological replicates (Supplementary Figure 2). However, no statistically significant results corroborate the existence of this peak. It has been demonstrated that E75 plays a role in molting, oogenesis and vitellogenesis of several insects^34–37^. It has been also suggested that E75 plays an important role in the interplay between the ecdysone and JH pathways^38,39^. In *Litopenaeus vannamei*, a crustacean decapod that is grouped with amphipods in Malacostraca crustaceans, E75 is positively regulated by ecdysone, regulates subsequent chitinase activities, and has molt stage-specific expression patterns^32^. The levels of EcR and E75 elevated significantly during vitellogenic stages I and II in the hepatopancreas of the crab *Oziothelphusa senex senex*^31^. Priya *et al*.^40^ showed that E75 is modulated through the molt process of *Fenneropenaeus chinensis*, and silencing the gene leads to a complete arrest of molting. However, other studies^41^ showed that E75 is marginally induced by ecdysteroids and presents constant levels of expression during the molt cycle and embryo development of the branchiopod *Daphnia magna*. The results obtained herein suggest that, similarly to other malacostraca crustaceans, E75 may play a role in female reproductive-related processes of *G*. *fossarum*, notably in vitellogenesis.

### BR

BR presented an up-and-down expression pattern, tending to a slight decrease in the later stages of the molt cycle. However, no deep modulations can be observed. BR is known to play a role in ecdysone and JH responsive processes such as metamorphosis^42,43^, vitellogenesis and oogenesis^42,44^ in several insects. Little is known about this gene in crustacean species, however it has been suggested that BR acts as a regulator of the action of methyl farnesoate (MF)^28^. Another study highlighted the importance of BR during ovarian development of *P*. *monodon*^45^. In that study, BR expression was more abundantly expressed in the ovaries than other tissues and injections of serotonin, progesterone and ecdysone all increased BR expression levels. This little information on BR function in crustacean species is complicated by the unknown evolutionary origin of this gene. Because there was no evidence of a BR gene ortholog in the non-insect and non-crustacean arthropod genomes, it has been suggested that BR was gained in the Pancrustacea^46^. Our results also highlight the limitations of using molecular information obtained from insects (or other phylogenetically distant species) for functional annotation of crustacean sequences (cross-species homology). The fact that the GFOSS putative BR sequence was not modulated during the reproductive processes opens several questions. The fact that this gene is a complex locus with many isoforms can be a limiting factor for the analysis. In order to investigate the role of different possible isoforms in *G*. *fossarum*, it would be necessary to test other BR-annotated sequences obtained by sequence homology and to check their expression patterns. The fact that the whole body of gammarids was used could have also diluted the response of this gene, and tissue specific modulations were therefore not detected.

### RXR

An intense peak at the post-molt stage A was observed for the RXR transcript. This peak was validated through the analysis of individual replicates, where a significant difference was observed between stage A and C2D1 (*p* value < 0.0001, Supplementary Figure 2). This stage lasts for only one to two days and is the shortest of the *G*. *fossarum* molting cycle. After this expression peak, RXR expression gradually decreased until the C1/C2 stage, stabilizing until the end of the cycle. This expression pattern suggests that RXR may be involved in ecdysis, since the expression peak takes place during this process. There are several studies on both RXR tissue-specific expression, and during the reproductive cycle of crustaceans. However, different trends are observed between studies, due to inter-species variability and to the existence of several RXR isoforms. For example, in the lobster *Homarus americanus*, RXR was more expressed in the mandibular organ (MO) than in other tissues^47^. In MO and in ovaries, an expression peak occurred in the intermediate stage of the reproductive cycle (secondary vitellogenesis), while for hepatopancreas the peak occurred only during the early stages^47^. In the decapod shrimp *Fenneropenaeus chinensis*, two isoforms of RXR were more expressed in both the gills and nerve cord, and their expression during the molt cycle using whole-body organisms showed a peak at late premolt and postmolt stages, suggesting a role in ecdysis^48^. Another decapod, *Portunus trituberculatus*, presented higher expressions in both the Y-organ (YO) and the ovaries, as well as a further expression peak in the YO during the post-molt stages^49^. The authors highlight that RXR has an effect on molting but its expression does not correspond to the ecdysone titers observed in their previous study^50^. Some studies already proposed that MF and RXR may have a specific signaling pathway independent of the ecdysone pathway in decapods. This pathway is thought to participate in Vtg gene expression and ovarian development^47,51,52^. Nevertheless, in line with most crustacean literature published, the results presented herein suggest that RXR might be involved in the ecdysone-mediated ecdysis process of *G*. *fossarum*.

### Expression following exposure to endocrine disrupting chemicals

In order to understand the susceptibility of E75, RXR and BR to EDCs, gammarids were exposed to three chemicals known to act on endocrine control of insect development. The chemicals were used at sub-lethal concentrations (PYR 5 μg/L, TEB 0.5 μg/L and PBO 150 μg/L) chosen based on previous experiments made in our laboratory, and therefore known to provoke reproductive impairments in female *G*. *fossarum*, particularly in vitellogenesis and oogenesis (Supplementary Figure 3).

Pyriproxyfen (PYR) is a potent juvenile hormone analog with insecticidal activity and little mammalian toxicity^53^, being very effective in inhibiting metamorphosis and embryogenesis in insects. Tebufenozide (TEB) is an analog for the molting hormone 20E. In lepidopteran species, the action of TEB results in the initiation of a precocious and incomplete molt (the new cuticle is formed before the shed of the old cuticle). This pesticide is not cleared by the organism, consequently inducing a continuous perturbation that maintains the expression of “early” response genes and the repression of genes normally expressed after the ecdysone peak for completing the molt^54^. Piperonyl butoxide (PBO) has no direct pesticidal activity of its own, but acts as a synergist of other insecticides by inhibiting some detoxification enzymes, namely the cytochrome P450 monooxygenases and some esterases^55^.

The durations of exposure were chosen based on the results presented above (Fig. 5). Our aim was to target the peaks of maximum expression for RXR in the beginning of the molting cycle (4 days, stage B) and for E75 in the onset of vitellogenesis (14 days, stage C2/D1). For RXR, since the stage A is very short, the stage B was selected for the first termination date. The nine day exposure (C1 molt stage) was intended to be an intermediate stage between the latter two in order to verify the occurrence of possible RXR/E75 inductions, as well as to confirm the trends between stages observed in the previous experiment (comparing the controls).

**Figure 6:**
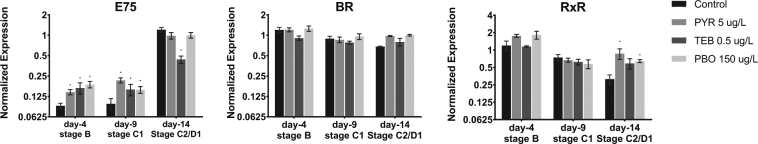
mRNA levels of E75, RXR and BR in control and exposed organisms for the three exposure times studied. Expression values of target genes were normalized to the expression of the reference gene EF. Each bar is the mean ± SD obtained from three experimental replicates. Each condition (control, PYR, TEB, PBO) contains mRNAs from five individuals pooled together. Statistical significance was accepted with fold changes > 1.5 and p-value < 0.05.

The values for the normalized expression in each condition, for the three target genes and at every exposure time, are shown in Fig. 6. Analyzing solely the control samples we can observe the same trends as before between the B, C1 and C2/D1 stages for the three genes analyzed, reinforcing the repeatability of the described expression pattern during female cycle. The more evident example is E75 expression, very similar between day-4 and day-9 experiments, with the peak appearing at day-14 when females are at their C2/D1 reproductive stage (fold change of 13 between day-4 and day-14). For both RXR and BR control samples, the expression pattern throughout the reproductive stage is also similar to the previous experiment, as shown in Table 3. Comparing control and exposed samples, some effects of contamination were observed, suggesting that the female endocrine system was disturbed upon exposure to the pesticides.

**Table 3:** Fold change expressions observed between different reproductive stages (B, C1 and C2/D1) in the two experiments performed.

		Experiment 1 Reproductive cycle	Experiment 2 Contaminant exposures
		fold change	fold change
E75	B/C1	1,27	1,07
	B/C2D1	11,68	13,30
	C1/C2D1	9,22	12,38
BR	B/C1	−1,19	−1,35
	B/C2D1	−1,32	−1,76
	C1/C2D1	−1,11	−1,31
RXR	B/C1	−1,43	−1,61
	B/C2D1	−3,18	−3,81
	C1/C2D1	−2,23	−2,36

To this respect, the **E75** transcript was the more responsive to contamination. At day-4 of exposure there was an increase in the order of 1.5 to 2-fold of E75 expression in all three contaminated conditions, a trend that repeated itself at day-9 (fold changes between 1.6 and 2.2). At day-14, transcript levels returned to normal in PYR and PBO exposures, while the TEB exposure led to a 2.8-fold decrease of E75 expression. The first increase in E75 expression means that the contaminants may induce, in a first phase, an over-expression of the “early” ecdysone-responsive genes in *G*. *fossarum*. These effects are concomitant with the role of this insecticide as an analog of the molt hormone 20E. However, the inhibition observed for the TEB exposed organisms at day-14 suggests that the ecdysteroid agonist effect of TEB is no longer taking place. Although in other species TEB is known to induce precocious molts through the continuous expression of the “early” ecdysone-responsive genes, in the studied species we have rather observed an inhibition of the early response-gene E75 after a 14-day exposure, which could reflect a general disruption of the normal ongoing of the female reproductive molting cycle.

E75 is a key molecule in the reproductive processes of arthropods, and since is an early ecdysone-responsive gene, is prone to be affected by interactions of chemical compounds with the ecdysteroid receptor which is at the upstream of the ecdysone regulatory cascade. In a recent study^56^, an Adverse Outcome Pathway (AOP) was developed for explaining the molecular events that lead to lethal molting due to ecdysone receptor agonism in arthropods. The mechanism proposed was based on the literature available for both insects and crustaceans. The expression of the E75 gene was considered the first key event of the molecular chain leading to the adverse outcome, reinforcing its potential to be used as a biomarker of endocrine disturbances.

Some effects were also seen in the expression of the **RXR** transcript at day-14 of exposure. PYR and PBO led to an over-expression of RXR at the C2/D1 stage (fold changes of 2.8 and 2.1 respectively). As discussed before, RXR and MF may have an ecdysone-independent regulatory pathway, which can explain the over-expression of RXR following exposure to the MF analog PYR. However, the possible mechanism that led to an induction of RXR provoked by PBO is not clear. It is known that this molecule does not have any insecticidal activity, but it inhibits the cytochrome P450 oxydases^57^, which participate in the ecdysone synthesis and inactivation pathways. If this occurs, we would rather expect an inhibition of RXR expression due to an inhibition of the 20E expression.

Concerning **BR** expression, some statistically significant modulations were observed, but with fold changes <1.5. This slight over-expression of this transcript at day-14 of exposure with PYR and PBO (fold changes of 1.43 and 1.47 respectively and p value < 0.001) follows the modulations observed for RXR, but establishing an eventual connection is difficult since the other early gene E75 was not modulated by the same exposure.

## Conclusion

Based on a RNAseq-derived transcriptomic database and phylogenetic analyses, we identified potential key players of the hormonal system of *G*. *fossarum*: the nuclear receptors E75, RXR and the regulator BR. Our analyses, in line with most of the information available in the literature, provided some relevant insights into the possible roles of E75 and RXR in vitellogenesis, oogenesis and ecdysis. Exposure to environmental concentrations of three EDCs provoked significant modulations in the levels of these transcripts. The role of BR in reproductive *G*. *fossarum* female processes was not elucidated in this study. Allied to the fact that this gene revealed less sensitivity to contamination than the others, BR is not proposed as a biomarker of EDC exposure in female *G*. *fossarum*.

These results provide the first elements for understanding the molecular mechanisms of endocrine regulation in gammarids. Despite calling for more mechanistic studies in order to unravel the roles of these genes in specific physiological processes, their susceptibility to being modulated by an exposure to EDCs was verified in this study. The rupture of the expression cycle of these genes is therefore proposed as a biomarker of EDCs effects in female *G*. *fossarum*. To our knowledge, our study is the first to analyze the impact of endocrine disruptors in ecdysone-responsive genes of *G*. *fossarum*.

## Methods

### Candidate selection and phylogenetic validation

#### Selection of *Gammarus fossarum* specific sequences

Given the importance of ecdysone in arthropod molting and reproduction, we focused on three ecdysone-responsive genes: RXR, EcR and BR. For each candidate, a BLASTP homology search was performed against our RNAseq-derived transcriptome database GFOSS^25^, using query sequences from phylogenetically close species obtained from the NCBI protein database. The EcR and BR query sequences used for blast were from the recently genome-sequenced amphipod *Hyallela azteca*, while the RXR sequence belonged to the crab *Eriocheir sinensis*. For this gene, decapods were the closest order to have sequenced RXR sequences in the NCBInr protein database. The BLAST search was conducted against both assembled contigs and unassembled reads databases. The top 3 blast scores with E-values inferior to E^−10^ and alignment length superior to 25% were chosen for the following analysis (Table 1).

#### Sequence alignment and phylogenetic analysis

For each candidate, a homologous sequence dataset was constructed using ortholog and paralog sequences from the same multigenic family as the candidate gene. The deduced *G*. *fossarum* sequences were aligned with other known sequences from a diversity of crustacean and insect species, obtained from GenBank (National Center for Biotechnology Information – NCBI) (listed in Supplementary Table 1).

Multiple sequence alignments and phylogenetic trees were performed in the SeaView software version 4.6.1^58^. Alignments were made using the Multiple Sequence Comparison by Log-Expectation (MUSCLE)^59^ program using default parameters. Phylogenetic tree model was determined using the IQ-TREE web server model selection^60^. Trees were built also in the SeaView, using the PhyML program based on the maximum-likelihood principle^61^, with the LG model with 4 substitution rate categories to estimate the gamma parameter shape, and 100 bootstrap replicates for branch support. Default settings were used for all other parameters.

### Gene expression studies

#### Sampling and maintenance of organisms

Gammarids were collected from the River Le Pollon in France (45°57′25.8′′N 5°15′43.6′′E) and acclimatized to laboratory conditions as previously described^33^. Organisms were collected by kick sampling using a net, and quickly transported to the laboratory in plastic buckets containing freshwater from the station. In the laboratory, organisms were kept in 30 L tanks continuously supplied with drilled groundwater (500µS/cm) and under constant aeration for at least 10 days. A 16/8 h light/dark photoperiod was maintained and the temperature was kept at 12 ± 1 °C. Organisms were fed *ad libitum* with alder leaves (*Alnus glutinosa*), previously conditioned for 6 ± 1 days in water. Sexually mature male and female organisms (selection based on the developmental stage of the embryos; well-developed juveniles in the female marsupium) were sampled for RNA extraction and gene sequencing protocols.

#### Selection of females at different phases of reproductive cycle

For gene expression studies, five females from each main and intermediate stages of the molt cycle (A, B, C1, C1/C2, C2, C2/D1, D1, D1/D2, D2, and final D2) were sampled. The selection of females at specific stages was determined through observation of characteristic integumental changes on the dactylopodite and protopodite during the molt cycle (described in^17^). Briefly, in *Gammarus fossarum* females, oogenesis and embryogenesis occurs simultaneously with the molting cycle. Six molt stages were previously defined^17^, A and B for postmolt, C1 and C2 for intermolt, and D1 and D2 for premolt. Organisms were incubated overnight at 4 °C in 300 µL of RNAlater, frozen in liquid nitrogen and immediately stored at −80 °C until total RNA extraction.

#### Laboratory exposure to endocrine disrupting chemicals

Coupled male and female organisms were sampled and isolated in a separated aquarium. Immediately after spawning, females were isolated and placed in 500 mL glass beakers (10 females per beaker, 3 beakers per condition). There were three conditions corresponding to three different exposure times: 4, 9 and 14 days. Solvent controls (uncontaminated drilled groundwater spiked with 0.005% acetone) were used to take into account any potential solvent effect in the interpretation of the subsequent gene expression analysis. Organisms were thus exposed to three EDCs: pyriproxyfen (PYR 5 μg/L), tebufenozide (TEB 0.5 μg/L) and piperonyl butoxide (PBO 150 μg/L). Stock solutions were prepared in acetone at concentrations of 100, 10 and 3000 mg/L for PYR, TEB and PBO, respectively, adding small aliquots of these stock solutions to the dilution water. Renewal of the media was performed manually every two days, and temperature was maintained at 16 °C throughout the experiment. At the end of the exposure (days 4, 9, and 14), five females from each condition were individually sampled, rapidly weighed, and incubated overnight at 4 °C with 300 µL of RNAlater. After incubation, samples were frozen in liquid nitrogen and stored at −80 °C until the RNA extraction protocol.

#### Total RNA extraction and cDNA synthesis

Whole-body organisms were disrupted with a TissueRuptor (Qiagen). For total RNA extraction, the RNeasy® Fibrous Tissue Mini Kit was used according to the manufacturer’s instructions (Qiagen). DNase I was used during the protocol to remove possible genomic contaminations. Total RNA was subsequently quantified using a NanoDrop 2000 spectrophotometer (Thermo Scientific), followed by a qualitative analysis using an Agilent Bioanalyzer 2100 RNA Nanochip (Agilent technologies, Santa Clara, CA). A quantity of 1 µg of RNA from each sample was converted into cDNA using the SuperScript® III First-Strand Synthesis System (Thermo Fisher Scientific) following the manufacturer’s protocol, and conserved at −20 °C until further analysis.

#### Gradient PCR amplification, cloning and sequencing

PCR was performed with a 50 µL mix of Millipore water containing 1 µL of cDNA, 2 µL of 10 mM dNTP, 2 µL of each primer at 10 µM, 5 µL of RT buffer, 3 µL of 25 mM MgCl_2_, and 0.5 µL of native Taq DNA polymerase (Thermo Fisher Scientific) (200U/µL). Primers (listed in Table 2) were manually designed, based on sequence alignments of *G*. *fossarum* deducted sequences with other arthropod sequences of the same gene.

The following PCR program was used (Biometra TGradient): initial denaturation step at 94 °C for 3 min, followed by 39 amplification cycles (denaturation 30 s at 94 °C; annealing 30 s at 56, 58.3, 59.7, and 62 °C; elongation 2 min at 72 °C). A final extension step was made with 10 min incubation at 72 °C. The PCR products were run on a 1% agarose gel (Supplementary Figure 1).

PCR amplicons were purified using the QIAquick®PCR Purification Kit (Qiagen). Purified fragments were inserted into pGEM®-T Vectors (pGEM®-T Vector System I, Promega), and transformed into DH5α competent cells. Clones containing target fragments were isolated and submitted for direct colony PCR verification using the following program: initial denaturation step at 94 °C for 10 min, followed by 40 amplification cycles (denaturation 30 s at 94 °C; annealing 30 s at 55 °C; elongation 1 min at 72 °C). A final extension step was made with 5 min incubation at 72 °C. The PCR products were run on a 1% agarose gel. To obtain high-purity plasmids, positive clones were purified with the QIAprep®Spin Miniprep Kit (Qiagen) following the manufacturer’s protocol. Samples were sent to Beckman Coulter Genomics (GENEWIZ®, UK) for sequencing.

#### Quantitative real-time polymerase chain reaction (qPCR)

For target genes, the same primers were used as for gene cloning (Table 2). Primers for reference genes, also listed in Table 2, were selected from previous studies that performed qPCR studies in *Gammarus* species^62,63^. Three reference genes were tested: Glyceraldehyde 3-phosphate dehydrogenase (GAPDH), 18 S RNA, and elongation factor 1 α (EF). In order to select a suitable reference gene, the stability of these three genes was assessed in cDNA samples from the physiological and exposure experiments. EF was considered as the most stable, presenting the lowest standard deviation for all Ct values obtained in the different samples, as shown in Table 2. The EF gene was therefore chosen to normalize expression data in both experiments.

For the quantitative PCR reactions, the iTaq™ Universal SYBR® Green Supermix (Biorad) kit was used. For the reactions, 25 ng (and 8.5 ng in the contamination experiment) of template cDNA and 300 mM of each primer were used, and the procedure was according the manufacturer’s instructions. PCR was performed in a CFX96 Touch™ Real-Time PCR Detection System (Biorad), using the following program: 30 sec at 95 °C, 40 cycles of 95 °C for 5 s, 60 °C for 30 s then a temperature increment was programmed for the melting curve (65–95 °C with 0.5 °C increments at 5 s/step). Samples for each condition were a pool of five biological replicates (RNAs extracted from the individual females were pooled together for the reverse transcription and subsequent qPCR). For the physiological experiment, we also analyzed the individual biological replicates in specific stages of the female reproductive cycle. The stages were selected based on the results from the pools, with the purpose of validating the observed peaks of expression. Therefore, for E75 we analyzed individuals from the C2D1, D1D2, and D2F conditions, while for RXR we analyzed individuals from the A, C2D1, and D2F conditions. All samples were analyzed in triplicate, and controls without DNA were run in every plate. Gene expression levels were analyzed using the relative quantification method (ΔCt)^64^.

### Statistical analysis

Statistical analyses for testing differences between conditions were performed with the GraphPad Prism Version 7.02 software, using unpaired t-tests corrected for multiple comparisons, according to the the Holm-Sidak method. Fold change was calculated using the 2^−ΔΔCt^ method normalized to the control condition. Significant differences were accepted when meeting both *p* < 0.05 and fold change > 1.5.

## Electronic supplementary material

Supplementary information
