# Supplementary material for: Identification, expression, and endocrine-disruption of three ecdysone-responsive genes in the sentinel species Gammarus fossarum
Source: Sci Rep. 2018 Feb 28;8:3793. doi: 10.1038/s41598-018-22235-7 (PMC5830573; doi:10.1038/s41598-018-22235-7)

**Identification, expression, and endocrine-disruption of three ecdysone-responsive genes in the sentinel species *Gammarus fossarum***

D. Gouveia^1,2^, F. Bonneton^3^, C. Almunia^2^, J. Armengaud^2^, H. Quéau^1^, D. Degli-Esposti^1^, O. Geffard^1^, A. Chaumot^1^*

^1^ IRSTEA, UR MALY, Laboratoire d’écotoxicologie, F-69625 Villeurbanne, FRANCE

^2^ Laboratoire Innovations technologiques pour la Détection et le Diagnostic (Li2D), Service de Pharmacologie et Immunoanalyse (SPI), CEA, INRA, F-30207 Bagnols sur Cèze, FRANCE

^3^ IGFL, Université de Lyon, CNRS UMR5242, Ecole Normale Supérieure de Lyon, Université Claude Bernard Lyon 1, 46 allée d׳Italie, F-69364 Lyon, FRANCE

***Corresponding Author**: Arnaud Chaumot

^1^ Irstea, UR MALY, Laboratoire d’écotoxicologie, Centre de Lyon-Villeurbannne, 5 rue de la Doua, CS 20244, F-69625 Villeurbanne Cedex, France

Tel: +33 (0)4 72 20 87 88

Email: arnaud.chaumot@irstea.fr

## Supplementary Table 1

GenBank accession numbers of the sequences used in sequence similarity searches and alignments/tree construction.

## Supplementary Figure 1

Fragments obtained after the temperature gradient PCR.

RXR -, BR -, E75 -, and EcR – correspond to negative (no DNA) controls.

L stands for ladder DNA.


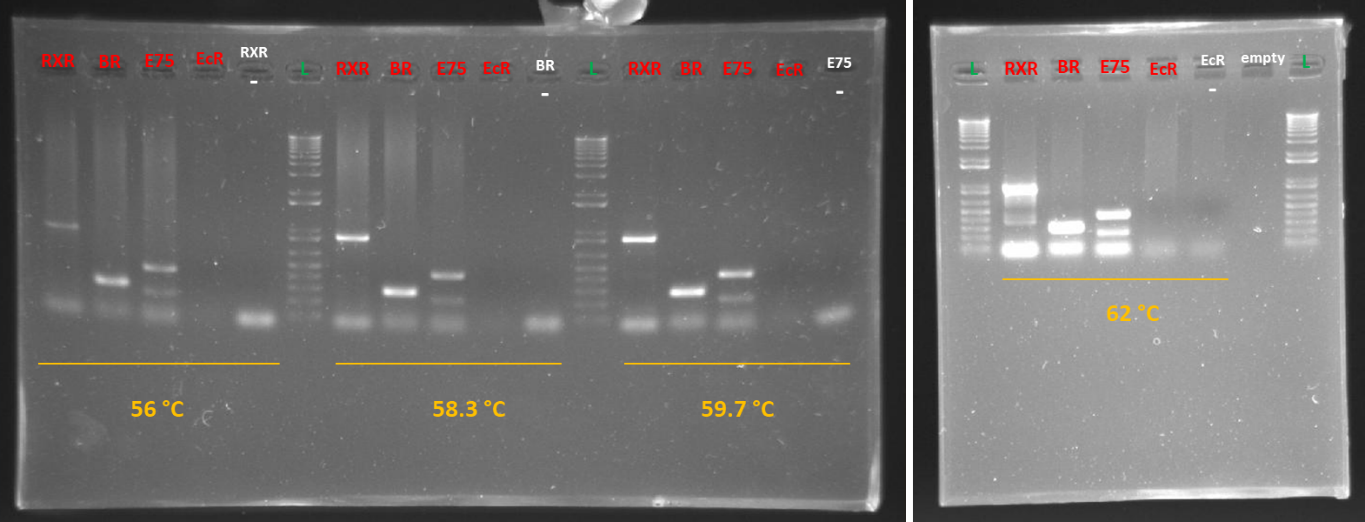


## Supplementary Figure 2

Data of individual replicates from the physiological experiment. Statistically significant differences were calculated through an unpaired t test with Welch’s correction. ****=p value<0.0001

## Supplementary Figure 3

Impact of tebufenozide and pyriproxyfen on oocyte development (bar graphs) and oocyte number (normalized to the size of the female) (boxplots) in *G. fossarum* females. Statistical differences were performed using a unilateral proportion comparisons test for the comparison of percentages (bar graphs), and non-parametric Mann-Whitney tests for the comparison of number of oocytes (boxplots) between control and contaminated samples. Significantly statistical differences were accepted at p<0.05.


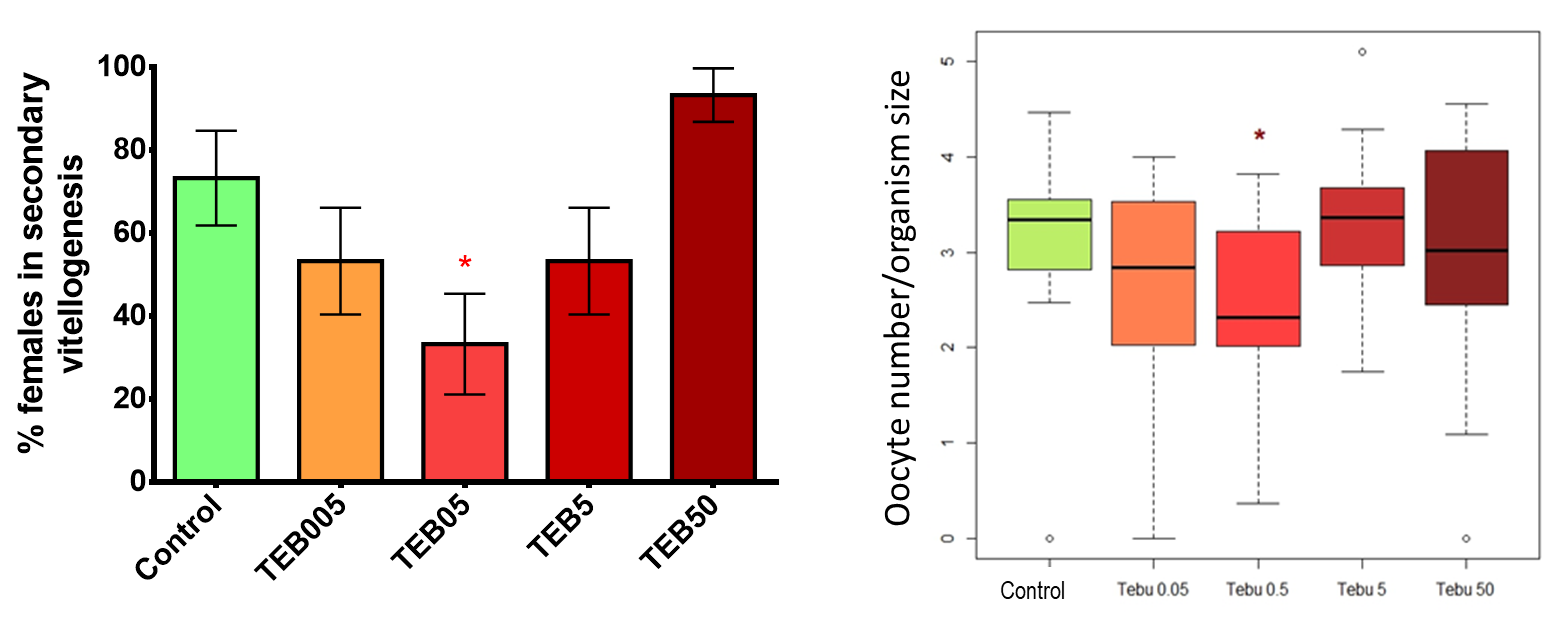


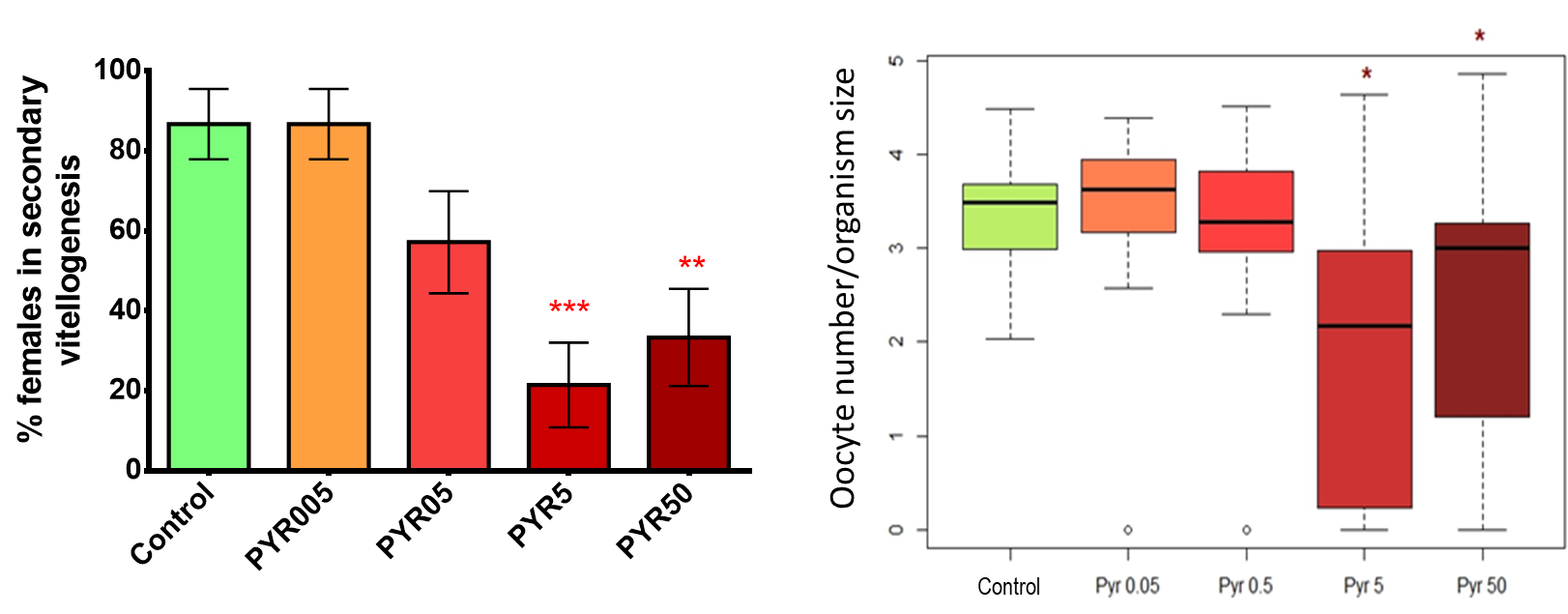

Supplement: Supplementary file 1 — Supplementary information [file 41598_2018_22235_MOESM1_ESM.docx]
